# Stable or Unstable? Evaluating the Strength Outcomes of 12-Week Resistance Training in Youth Judo Athletes

**DOI:** 10.3390/sports12120352

**Published:** 2024-12-19

**Authors:** Nemanja Stanković, Dušan Stupar, Aleksandar Ignjatović, Nikola Milošević, Nebojša Trajković

**Affiliations:** 1 Faculty of Sport and Physical Education, University of Niš, 18000 Niš, Serbia; nemanjastankovic84@hotmail.com (N.S.); milosevicn@yahoo.com (N.M.); nele_trajce@yahoo.com (N.T.); 2 Faculty of Sport and Psychology, Educons University, 21000 Novi Sad, Serbia; 3 Faculty of Education, University of Kragujevac, 35000 Jagodina, Serbia; aleksig79@yahoo.com

**Keywords:** unstable, resistance training, young athletes, judokas, strength

## Abstract

**Background**: The purpose of this study was to compare the effects of resistance training on stable versus unstable surfaces on strength performance in young judokas. **Methods**: The study included 18 young judokas (age: 13.2 ± 1.2 years) with 4.7 years of training experience assigned to either the URT (unstable resistance training) group or the STG (stable resistance training) group. Both groups performed the bench press and squat exercise for 12 weeks (3 sessions/week, 30–40 min each), with one group performing on the stable surface and the other on the unstable surface. The tests included the 1RM bench press and squat, maximal power output during bench press and squat (Pmax), abdominal strength test (AB60), and the standing long jump (SLJ). **Results**: Statistical analyses revealed a group × time interaction for AB60 (*p* < 0.02) in favor of the URT group. Significant main effects of time (*p* < 0.01) for the 1RM bench press, 1RM squat, bench press power, AB60, and SLJ were found. There were no significant effects for squat maximal power in both groups (*p* ˃ 0.05). **Conclusions**: Both unstable and stable resistance training effectively improved maximal strength and performance in adolescent judokas, with unstable training offering additional benefits in abdominal strength.

## 1. Introduction

Judo training in children and adolescents can enhance motor learning, increase anaerobic capacity, flexibility, strength, and power; improve body composition; and provide an effective strength and conditioning base for other sports [1]. As youth sports become increasingly popular [2,3], young athletes, their parents, and coaches are looking for the best ways to gain a competitive edge. Muscular strength and power are essential for success in many sports, especially in combat sports, such as judo. It is clear that stronger and more powerful young athletes will perform better and, more importantly, develop a higher and stronger base for future development. The safety and efficacy of resistance training in youths has previously been well documented [4,5]. While there are recommendations for strength and conditioning for senior judo athletes [6,7], the recommendations for judo strength training for young athletes remain scarce. Judo coaches consider motor fitness preparation as an important item for success in competition, together with technical, tactical, and psychological preparation [8]. Keeping this information in mind, a strength and conditioning program for young judokas needs to be as time-saving as possible, of lower volume and intensity, in order to be more easily combined with technical and tactical training.

In order to maintain balance under external forces produced by the opponent, high core muscle activation is necessary. It is often difficult to target core muscles effectively with traditional stationary exercises, so additional or specially designed sport-specific exercises are welcome. In the last decade, unstable training has frequently been used in training because it is believed that unstable environments create an increased level of muscle activation [9,10,11]. Moderate instability typically occurs in strength training scenarios involving free-weight lifts performed on stable surfaces, which challenge balance and control to a manageable extent without significantly reducing force output. Greater instability levels are introduced when strength training is conducted on unstable surfaces, as these conditions amplify center of pressure excursions and movement unpredictability [11]. It has been suggested that even moderate instability can produce optimal results not only by allowing sufficient stress on the body while aligning with recommendations for low- to moderate-intensity resistance training for children and adolescents [10,11,12] but also with injury prevention and rehabilitation programs [11,12].

Several research studies [13,14,15,16,17,18] have demonstrated that an unstable exercise program elicits a greater effect on muscle strength than has been identified as a prerequisite for success in several sports [19,20]. However, a systematic review and meta-analysis [21] failed to show the significant advantage of the unstable surface in adolescents and young adults, suggesting that the use of unstable surfaces as compared with stable surfaces during strength training will not enhance the performance on stable surfaces. Although the effects of unstable surfaces during resistance training have been widely studied in general athletic populations, there are a limited number of studies involving adolescents [22,23,24] and no studies involving children that examined the effects of unstable resistance training programs. Judo is a dynamic sport. However, good stability in judokas may be crucial for maintaining posture, executing techniques effectively, and preventing injuries. However, the potential benefits of training on unstable surfaces have not been explored in the context of youth judokas. By investigating how unstable surfaces affect muscle activation and overall strength performance in young judokas, this research could offer valuable guidance on integrating unstable surface resistance training into judo-specific conditioning programs. Therefore, the aim of this study was to compare the effects of resistance training on stable versus unstable surfaces on strength performance in young judokas. It was hypothesized that 12 weeks of unstable resistance training would provide significantly greater training gains than stable training in strength performance tests.

## 2. Materials and Methods

### 2.1. Participants

A total of 29 participants initially took part in the study. In the beginning, 15 young judokas were recruited for the unstable resistance training group (URT), and 14 for the stable resistance training group (SRT). The allocation of participants into groups was conducted using an online randomization software, namely www.randomization.com, to ensure a randomized and unbiased distribution. Children with diagnosed injuries (*n* = 2) and children that failed to participate in 85% of the sessions (*n* = 4) were excluded from the study. Also, children that failed to conduct at least one of the evaluation tests were excluded from the study (*n* = 5). Two overexertion-related muscle disorders occurred throughout the study period, but none of them as a result of the intervention. Due to increased school obligations, the common cold, temporary or permanent dropouts from regular judoka training, or other private reasons, nine participants failed to participate in more than 85% of the sessions or the final testing and were excluded from the analysis. Finally, the research was completed on a sample of 18 participants (11 in the URT group and 7 in the SRT group) who underwent the entire assessment and intervention program. All of the participants were young judokas involved in organized training (five days per week) and competition in their age category. Their average organized training background in the club was 4.7 years (ranging from 1.1 up to 8 years). None of the participants involved in this study had taken part in organized and programmed resistance training with additional free weights before. Two weeks of familiarization (4 sessions) were conducted in order to introduce the proper bench press and squat technique to all the participants. Their mean age was 13.2 years, mean height was 166 cm, and mean weight was 55.4 kg. The URT consisted of 11 children whose mean age was 13.2 years; mean height was 165.7 cm, and mean weight was 53.2 kg. The SRT consisted of 7 young judokas whose mean age was 13.2 years, mean height was 166.4 cm, and mean weight was 59 kg. The maturity level of all participants was evaluated, and the years to peak height velocity for the unstable group was −0.7 ± 0.5 years, while for the stable group, it was −0.6 ± 0.7 years, with no significant differences observed between the groups. Of all the girls initially engaged in the study, only one from each group remained for the final testing. Given that the participants were children, it is important to note that differences between genders at this developmental stage are not always as pronounced. Moreover, while the sample sizes of the two groups differed in number due to lower study enrollment in the stable resistance training group, dropouts, and failing to conduct some of the testing, there were no significant between-group differences for age, height, and weight (independent samples *t*-test).

This study was approved by the Ethics Committee from the Faculty of Sport and Physical Education, University of Nis, according to the revised Declaration of Helsinki (04-1847/2; date of approval 26 November 2020). Written parental/guardian consent was obtained at baseline, and verbal child assent was obtained at each assessment time point. The children were asked if they were willing to complete the tasks, and if they were not willing to complete them, they were excluded from participation.

### 2.2. Procedures

All of the testing sessions took place in a sports hall and gym, which are part of a laboratory for multidisciplinary studies. Prior to testing, the participants warmed up for approximately 10–15 min of submaximal intensity aerobic activity and short bouts of dynamic muscle stretching. The aerobic activity included light jogging to gradually increase heart rate and prepare the body for more intense physical exertion. Afterward, dynamic stretching exercises were performed to enhance muscle flexibility and joint mobility, which are essential for injury prevention and optimal performance during the tests. The dynamic stretches included leg swings, arm circles, high knees, butt kicks, and walking lunges. These exercises were selected to target the major muscle groups used during the testing, including the legs, hips, and upper body, and performed in a controlled manner to avoid overstretching or fatigue before the testing began. After that, the following strength performance tests were conducted: the 1RM bench press, 1RM squat, power during maximal speed of movement during the bench press and squat with a barbell, the abdominal muscle test (AB60), and standing long jump (SLJ). All testing was conducted over two testing sessions during the same week, within regular training intervals. All of the participants were randomly assigned to different groups, so while one group performed the bench or squat testing, the other group performed the SLJ and abdominal muscle test (AB60). The same researchers supervised both the initial and final testing. All researchers had a background in testing in sport and physical education and several years of experience with physical evaluation testing of children and judokas.

Participants underwent familiarization sessions for all tests used in the study, with particular emphasis on the 1RM testing to ensure safety and proper technique, given their age. Familiarization with the standing long jump test and the 60 s sit-up test was conducted in a single session, as these are standardized school tests that participants were already familiar with through their physical education curriculum. A previous study [25] demonstrates that healthy children can safely perform 1RM strength tests, provided that appropriate procedures are followed. Emphasis was placed on squat exercises, achieving a knee angle of 90° during the squats, proper technique (i.e., controlled movements during the concentric and eccentric phase), proper spine posture, and breathing. During the familiarization protocol, participants followed a structured approach to ensure proper technique and safety [25]. For the squat exercise, participants were instructed to position their feet shoulder-width apart, with toes pointing slightly outward for balance and stability. In the bench press, participants were required to maintain five contact points (head, upper back, buttocks on the bench, and both feet flat on the floor) and lower the barbell to touch the chest lightly before pressing it back to full extension, with controlled movement throughout the eccentric and concentric phases. Loads were kept light, corresponding to approximately 30–40% of the participants’ estimated one-repetition maximum (1RM), to allow for controlled movement and focus on technique. Participants performed 2–3 trials per session to refine their technique, with the number of repetitions per trial decreasing as they approached their estimated 1RM. Progression during the familiarization was controlled, with an increase in weight only after participants demonstrated proper form and confidence. This approach ensured that all participants were adequately prepared for the actual testing, minimizing potential risks and standardizing the execution of exercises across sessions. After the completion of proper technique familiarization sessions, the participants had a familiarization session specifically designed for the testing procedures for the Fitrodine dynamometer evaluation. All familiarization sessions were conducted over two weeks prior to testing as a part of regular judoka training. All of the participants had the same number of familiarization sessions before the testing procedures and were instructed not to engage in exhausting exercise outside of their regular training routine for a period of 48 h prior to testing. They were warned to refrain from eating or drinking energy drinks for two hours prior to the testing. The testing procedures were well tolerated by the participants. No complaints of severe muscle soreness were reported. Additionally, any questions regarding the training and testing procedures were answered.

### 2.3. Tests

Abdominal muscle strength test (AB60). Sit-ups in 60 s is a test often used as an indication of abdominal strength. The participant should perform as many sit-ups as they can in 60 s. The test starts in a supine body position with fingers interlocked behind the head and knees bent at approximately 90°. The participants were asked to flex their trunks up to the point when their elbows touched their knees. For each sit-up, the back must return to touch the floor. A researcher secured the feet to the floor to prevent sliding and lifting of the legs. The result is the total number of correctly performed sit-ups within 60 s. The test was performed twice with 2 min rest in between. The literature indicates excellent ICC reliability scores (0.96) for this sit-up test in school children and adolescents [26]. Moreover, the reliability coefficient (ICC) for the test in the current research was r = 0.92.

Standing long jump test (SLJ). The standing long jump was shown to provide a valid and reliable assessment of power among young children [27] and adults [28]. The ICC in the current SLJ tests was r = 0.96. The participants were positioned on a tatami surface with both feet behind a line, according to test instructions. By bending the knees while freely swinging the arms, the participant jumps forward as far as possible. The distance is measured from the take-off line to the rearmost heel. Three trials were performed with a 2 min rest between trials, and the best score was taken for further analysis. The score for each test was recorded to the nearest 1 cm using a tape measure. One repetition maximum test (1RM). The load for 1RM was determined for each participant in the bench press and squat exercises using standardized protocols designed for children and adolescents. [25]. Prior to the 1RM test, participants completed a progressive series of warm-up sets: 6–10 repetitions with a light load (~30% of estimated 1RM), followed by 4–6 repetitions at ~50%, 2–4 repetitions at ~75%, and a single repetition at ~90% of the estimated 1RM. These warm-up trials allowed participants to prepare physically and mentally while minimizing the risk of fatigue or injury. During the 1RM test, the tempo of each lift was not strictly controlled, but participants were instructed to perform each movement in a controlled and deliberate manner, ensuring proper technique and maintaining consistent movement throughout the full range of motion. The 1RM was recorded as the maximum resistance that could be lifted once, through the full range of motion, with proper form. Participants were allowed 5–8 attempts to determine their 1RM, with rest periods of 3–5 min between trials. Incremental increases in load (ranging from 2.5 to 7 kg) were applied based on the participant’s performance and the effort required during each lift, with smaller increments used as the participant approached their maximum. Failure was defined as the inability to complete the full range of motion or to maintain proper technique on two consecutive attempts. The ICC for the 1RM tests was r = 0.91 and 0.88 for the bench press and squat, respectively.

Muscle power output test. Muscular power outputs for the bench press and squat exercises under stable conditions were measured by means of the Fitrodyne dynamometer (Fitronic, Bratislava, Slovakia), a device validated and found reliable for muscle power measurement by Jennings et al. [29]. The FitroDyne operates by attaching to the barbell with a nylon tether, recording the displacement rate at a frequency of 100 Hz, and calculating the velocity and power output during the lifting motion. During each attempt, the participants were instructed to accelerate the barbell as fast as possible during the concentric phase of motion, during which the peak power and velocity of movement were measured by means of a computer-interfaced Fitrodyne attached to the barbell via a tether. For the bench press, participants used a prone grip to lower the barbell to their chest in a controlled manner during the eccentric phase, followed by a maximal push during the concentric phase until full elbow extension. For the squat, the barbell was positioned across the shoulders, and participants descended until the hips were below the knee joint, using a bench as a reference to ensure consistent depth across trials, before ascending rapidly to full knee extension. Each movement was performed without pauses between phases or bouncing of the barbell. Testing loads were set at 30% and 50% of 1RM, as these intensities have been identified to optimize power output in adolescents. A warm-up with submaximal loads preceded the test to ensure proper technique and readiness. Participants performed three repetitions for each load, with self-selected rest intervals capped at 90 s, depending on the perceived exertion of the preceding trial. Peak and mean power outputs were recorded for each lift, with the highest power value from the trials used for analysis. The ICC for the maximum power tests was r = 0.92 and 0.84 for the bench press and squat, respectively.

### 2.4. Training Program

The 12-week intervention program was conducted in a gym at the Faculty of Sport and Physical Education and integrated into the athletes’ regular judo training sessions. Each session, supervised by the head coach and researchers, combined judo training and additional resistance training under stable or unstable conditions. During the program, there were no judo competitions. Emphasis was on technical improvement (uchi komi and nage komi), which is also used to improve aerobic and anaerobic fitness [30] and strength training under stable or unstable conditions. Judo training followed a structured routine adapted from Branco et al. [31] and Fukuda et al. [32], beginning with a 15 min warm-up that included interval running, static and displacement shadow techniques (uchi-komi), and ukemi (falling techniques). This was followed by 25–30 min of technical practice focused on uchi-komi for te-waza, ashi-waza, koshi-waza, and sutemi-waza, performed in line and with displacement, as well as nage-komi (throwing drills). The session progressed with 30 min of match simulations, including groundwork (ne-waza) and standing matches (randori) with different partners, and concluded with a 10 min cool-down involving stretching and breathing exercises. Resistance training was performed after the judo session, focusing on the bench press and squat exercises under stable or unstable conditions. For unstable conditions, Bosu balls and Swiss balls were used, with the bench press performed on a Swiss ball and the squat on a Bosu ball. The resistance training protocol involved 6 sets of 8–12 repetitions at 50% of the previously determined 1RM during the first 6 weeks and 60% of 1RM from weeks 7 to 12, with 90 s of rest between sets and 3 min between exercises. Participants alternated the starting exercise (bench press or squat) in each session to prevent order bias. Sessions began with a 10 min dynamic warm-up and concluded with a 10 min stretching routine. All of the participants had the same weekly training routine. The only difference between the groups was in the additional resistance training program with or without unstable surfaces. Researchers monitored all resistance training sessions to ensure proper technique and effort.

### 2.5. Statistical Analysis

Descriptive statistics (means ± standard deviation) were obtained for anthropometric, weight and strength, and performance test variables. The Levene test was used to check the homogeneity of variance among the pretraining variables, and the normal distribution of data was confirmed by the Shapiro–Wilk test. Repeated measures analyses of variance (2 × 2 ANOVAs) with a within-factor variable (time) with two levels (pre and post intervention) and a between-factor variable (the treatment group) with two levels (URT and SRT) were used to evaluate the intervention effect on the strength and performance tests. In cases in which significant differences in the interaction Group × Time were detected (*p* < 0.05), a Bonferroni procedure was used to identify group differences. Effect sizes (ES = mean change/SD of the sample scores) were also calculated and reported. To determine the magnitude of the response to both training programs, we analyzed the effect size (ES) using descriptors to indicate the changes (small < 0.41, moderate 0.41 to 0.7, or large > 0.7). In addition to this testing, for each variable, the percentage of the difference in the change in scores between the URT and SRT groups from the pre- to the post-test was calculated. All of the statistical tests were performed using the program SPSS version 17.0 (SPSS, Chicago, IL, USA). Significance was set at *p* < 0.05.

## 3. Results

**Figure 1:**
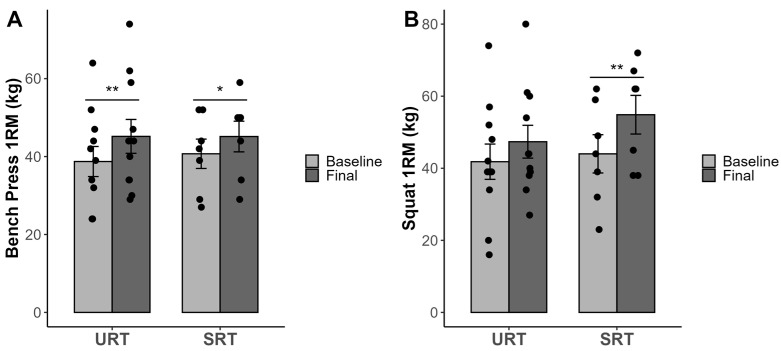
1RM bench press (**A**) and squat test results (**B**). *, ** Significant difference between pre- and post-measurement *p* < 0.05 and *p* < 0.01, respectively. Abbreviations: URT = unstable resistance training group; SRT = stable resistance training group.

**Figure 2:**
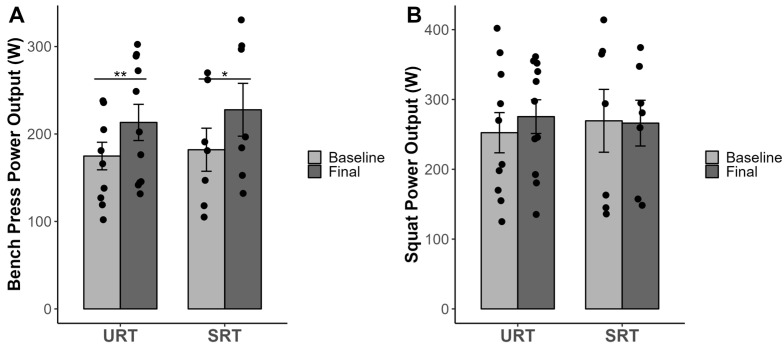
Maximal power output during bench press (**A**) and squat (**B**). *,** Significant difference between pre- and post-measurement *p* < 0.05. and *p* < 0.01, respectively. Abbreviations: URT = unstable resistance training group; SRT = stable resistance training group.

**Figure 3:**
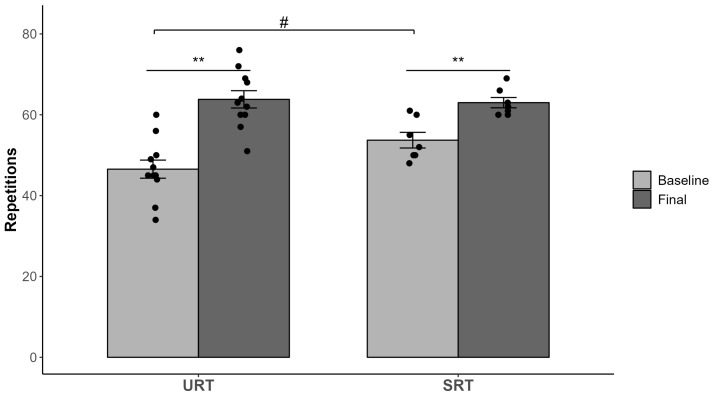
Abdominal muscle strength test (AB60) results. Significant differences within groups (Baseline vs. Final) are indicated by ** *p* < 0.01. Between-group differences at the baseline are denoted by # *p* < 0.05. Abbreviations: URT = unstable resistance training group; SRT = stable resistance training group. Bars represent mean ± SE (standard error).

The results of the study are presented in Table 1. Additionally, the data are visually represented in Figure 1, Figure 2 and Figure 3, highlighting the individual scores and differences. A one-way ANOVA test was conducted, and no significant differences (*p* > 0.05) existed at baseline between the treatment groups (URT and SRT) for the tested variables.

### 3.1. Muscle Strength: The Bench Press and Squat 1RM and Power

The repeated measures 2 × 2 (group × time) ANOVA showed that there were no significant interactions (group × time) in 1RM for the bench press (F (1,16) = 1.039, *p* = 0.323, partial eta squared = 0.06) and for the squat (F = 0.744, *p* = 0.401, partial eta squared = 0.06). However, there were significant effects in time (F = 29,976, *p* =0.0001, and partial eta squared = 0.65, and F = 15.075, *p* = 0.001, and partial eta squared = 0.49 for the bench press and squat, respectively). Both experimental groups (URT and SRT) achieved significant increases in the 1RM bench press, *p* < 0.001 and *p* = 0.032, respectively. For the 1RM squat, only the SRT group achieved statistically significant pre- and post-treatment changes, *p* < 0.001, while for the URT group the changes were non-significant, *p* = 0.076.

There were no significant interaction effects (group × time) for power output for the bench press (F = 0.173, *p* = 0.683, partial eta squared = 0.01) and for the squat (F = 0.647, *p* = 0.433, partial eta squared = 0.04). There were significant effects in time for power in the bench press (F = 22.974, *p* = 0.001, partial eta squared = 0.59) but not for the squat (F = 0.365, *p* = 0.556, partial eta squared = 0.02). Both experimental groups (URT and SRT) achieved significant pre-post treatment changes in the bench press power of *p* < 0.001 and *p* = 0.047, respectively. Neither of the groups achieved an increase in the squat maximal power test, *p* > 0.05 (Table 1).

### 3.2. Muscle Performance Strength Tests

Significant interaction effects (groups × time) were observed for the AB60 performance test (F (1,16) = 7.024, *p* = 0.017, partial eta squared = 0.31) and for the time (F = 77.665; *p* < 0.001; partial eta squared = 0.83). Pre- and post-treatment changes for both groups are presented in Figure 3. Both experimental groups (URT and SRT) achieved significant increases in AB60 performance (*p* < 0.01).

For the SLJ, no significant interaction effect (groups × time) was observed (F (1,16) = 0.056, *p* = 0.816). However, there was a significant time effect (F = 17.463; *p* < 0.001; partial eta squared = 0.52). Both experimental groups (URT and SRT) achieved significant increases in the SLJ performance of *p* < 0.010 and *p* = 0.031, respectively.

## 4. Discussion

The purpose of this study was to investigate the effects of additional resistance training on unstable vs. stable surfaces on maximal strength and performance among adolescent competitive judokas. The main findings indicated that both URT and SRT improved maximal strength and performance, but URT led to greater gains in abdominal strength. Increases in strength performance tests over a 12-week training period were noted for the abdominal muscle performance test (AB60), lower body explosive power (SLJ), 1RM bench, 1RM squat, and maximal power during bench press (PmaxB). There were no significant differences found for training-induced improvements between the pre- and posttest in maximal squat power output (PmaxS). Therefore, the hypothesis was partially confirmed, as 12 weeks of URT led to significantly greater gains in abdominal strength but did not result in superior improvements in muscular power and other performance tests compared to SRT.

The only significant differences found for training-induced improvements between the training groups in the pre- to post-test were determined for the AB60 test in favor of the URT group. Larger effectiveness of unstable training on abdominal muscle strength could be explained by the greater extent of abdominal and paraspinal muscle activation under unstable compared to stable conditions [11,21]. Increased abdominal muscle activity and increased perceived exertion were reported when performing chest presses and squats on a physioball [16,17]. A higher degree of instability when performing the squat resulted in approximately 20%–30% greater, as well as a prolonged activation of the abdominal and paraspinal muscles [15]. Also, Freeman et al. [33] reported that ballistic dynamic push-ups required greater muscle activation and spinal loading when push-ups were performed on an unstable surface (basketball balls). Therefore, the findings suggest that instability devices may provide a greater training stimulus and high core muscle activation even with less intense limb exercise. Possible explanations for differences could be that stable conditions often emphasize limb movements or large compound exercises, which, while engaging the core for stability, may not effectively target deeper stabilizing muscles. In contrast, instability devices create an unstable environment that necessitates continuous adjustments, thereby promoting greater activation of these hard-to-target muscles, which are essential for balance and postural control. Additionally, it is important to stress that the URT group had slightly worse results on the initial measurement and therefore needs more space for improvement in time-limited tests (60 s).

Improving abdominal strength would allow a judoka to increase the ability to generate and maintain force throughout a fight [34]. Moreover, improved abdominal strength might contribute to postural performance [35] and even judo performance, as it would facilitate the transmission of forces generated by the limbs during specific judo techniques [36].

Some previous studies had, to some extent, contradictory results regarding the effects of an unstable training program on abdominal muscle strength. A few studies failed to determine the advantages of an unstable program on the abdominal strength test [37,38]. The first study [37] included a full-body training program, which led to similar improvements under unstable and stable conditions after 10 weeks of training. The second-mentioned study by Cowley et al. [38] included only the upper body bench press without lower body unstable exercise and only 3 weeks of intervention. On the other hand, Kimble and Behm [39] demonstrated an increase in the number of sit-ups performed (8.9%) as the result of an unstable resistance training program compared to the results of a stable training group after seven weeks. Our study led to an even larger increase (URT 37% vs. SRT 17%) in the abdominal strength test AB60 after a longer (12-week) training intervention, which included the bench press and squat with additional resistance on unstable surfaces. Our findings indicate that a longer intervention program affecting core muscles by exercising both the upper and lower body under unstable conditions could be effective for the increase in abdominal muscle strength performance.

The torso and arms are generally the parts exposed to the greatest “instability” applied further up the kinetic chain, even on stable surfaces. This is why URT could be more useful when training the “core” and upper body rather than the lower body [40]. The results of previous studies included in the meta-analysis [21] suggest that the use of unstable as compared to stable surfaces during strength training is not recommended in healthy adolescents and young adults if the goal is to enhance performance measures such as maximal strength and power on stable surfaces. This recommendation is rooted in the principle of specificity, as training on unstable surfaces may limit the load that can be lifted, reducing the potential for neuromuscular adaptations that directly transfer to stable-surface tasks.

Studies that tried to determine the effects of unstable training on lower body muscle power results mostly depended on the study sample training background. Some studies [40,41], which include trained adult athletes, suggest that lower body unstable training attenuates jumping performance. Despite studies that include inexperienced resistance training participants, unstable resistance training may be considered as effective as traditional stable resistance training [36,39,42]. Among young athletes, unstable training as compared to stable training achieved mostly similar improvements on the physical performance tests of lower limbs [22,23,24]. Additionally, with the use of plyometric exercises, unstable training appeared to be less effective for increasing CMJ height compared to stable conditions [23]. These findings indicate that the specific nature of exercise selection should be considered.

The present study observed no significant changes in SLJ between the unstable and stable groups. Both groups had an almost identical improvement of 5% following 12 weeks of resistance training. However, there was a slight difference in the maximal strength test (1RM) between the groups. There were significant differences in effects between groups in the 1RM squat. However, the stable training group had significant pre- and post-treatment changes compared to the URT group that showed no significant changes. The principle of training specificity and testing conditions on stable surfaces may explain why training on stable compared to unstable surfaces led to greater improvements. However, in the 1RM bench press, there was no training-specific transfer on testing on the same surface. There were no significant differences in effects between stable and unstable conditions. Contrary to training specificity, the URT group improved more than the SRT group, 18% and 11%, respectively. Both experimental groups had the same external resistance, which, with additional instability, resulted in increased intensity of the exercise for the unstable group. This may be the principal reason for the better results of the unstable training group on the strength test expressed as 1RM on a stable surface. A study by Sparkes and Behm [42] found no training effects in any measure but suggested a tendency for unstable training to be more efficient for maximum voluntary isometric contraction in the bench press exercises. They used the same relative resistance for both surfaces (10RM), but the 1 repetition maximum on an unstable surface was lower compared to the stable surface, which implied that the group that trained on unstable surfaces used lower loads during exercise and achieved similar improvements.

Still, there is the open question of why the same external load during the squat did not result in increased intensity and better results in the unstable group, as it did during the bench press. Additionally, a previous study conducted by the same research group [43] among healthy young adults resulted in a significant improvement in the 1RM squat but not in the 1RM bench press after an unstable training program.

We could speculate that young athletes without previous experience in squat exercises improved more on the 1RM squat on stable surfaces because they had more time to learn and master proper technique while training on the same surface. This was the first time that they performed squat exercises with additional load, and despite four familiarization sessions, the group that performed training on stable surfaces had much more time to master the squat technique compared to the group that performed on unstable surfaces. Regarding power output, there were no significant effects over time on the maximal power output in the squat. Both groups failed to improve maximal power output during the squat. The stable group even had a minimal decrease (−1%). Training for power was not the goal of the training program, so it might explain the lack of a positive effect during more complex multipoint movements such as the squat, especially when performed as fast as possible. Considering that the training program was conducted with young, previously inexperienced resistance training athletes and that training was focused on proper technique and posture without explosive movements during exercise, it could explain why the program had no effect on maximal power movement during squat testing.

Improvements in 1RM on the stable and unstable surfaces following 12 weeks of training ranged between 11% and 17% (ES 0.43–0.48) for the bench press and 18% and 26% for the squat (ES 0.46–0.80). These improvements are in agreement with the previously observed improvement after training under unstable conditions, with small to medium effects detected for measures of maximal strength in adolescents [21]. However, in addition to the abovementioned differences in effects, we found no significant differences between stable and unstable conditions for measures of maximal power output during the bench press. Both groups showed similar improvements.

The obtained results demonstrate that a greater degree of stress (unstable surface) does not lead to greater systematic strength improvements in young, adolescent judokas. Furthermore, a comparison of the obtained results with previous research [44] on a sample of highly trained competitive judokas (mean age of 21 ± 1.3 years) shows that the performance of a resistive exercise on an unstable surface among judokas may also be influenced by age and training experience. It can be concluded that the application of unstable resistance training compared to stable resistance training has limited additional effects on measures of muscle strength in adolescent judokas.

Only a limited number of studies examined resistance training intervention effects in young, previously trained teenage athletes. Longitudinal studies with unstable training among young athletes are even more difficult to find. One of the advantages of this study is the administration of the selected exercises within the scope of a regular five-day training program each week, with the additional effort to implement resistance training exercises among young athletes without previous experience in resistance training and proper lifting technique.

On the other hand, there are a few limitations of the present study as well. The lack of a control group is acknowledged as the largest weakness of the experimental design. However, it would be unfair to select some young athletes for the control group without resistance training and explain to them that they need to serve as a control. Additionally, there were a considerable number of dropouts from the initial experimental design. Two participants were excluded due to minor injuries unrelated to the experimental design, which prevented them from participating in the program and maximal testing safely. Everyday training was difficult to accomplish and combine with regular school and private obligations of teenagers, so nine participants failed to complete the necessary regular training and testing. It also led to an unequal number of participants in the intervention groups (11 versus 7).

Therefore, the use of unstable surfaces during resistance training among young teenage athletes can only partially be recommended. Further research is needed to determine the effectiveness of other forms of resistance exercises on young teenage athletes and compare them with a control group in order to separate the effects of training from the effects of growth and development.

## 5. Conclusions

Both unstable and stable resistance training effectively improved maximal strength and performance in adolescent judokas, with unstable training offering additional benefits in abdominal strength. These findings suggest that incorporating instability into training programs may provide targeted advantages, particularly for developing core strength. However, it is important to note that unstable training does not outperform stable training in overall power and performance. While the scientific literature consistently highlights the benefits of regular judo practice in enhancing muscle strength among children and adolescents, our results underscore the potential of supplementary resistance training to further improve maximal strength. Specifically, unstable resistance exercises can serve as an effective approach to enhance core muscle activation and abdominal strength. Nonetheless, coaches are encouraged to investigate the optimal integration of unstable resistance exercises into training regimens, considering that young judokas develop postural strategies early in their practice to maintain stability across diverse conditions.

## Figures and Tables

**Table 1:** Effects of a 12-week additional resistance training program on all performance variables for both groups. Pre-test and post-test values are presented as means (±SD).

Variable (Unit)	Group	Pre–Test	Post–Test	Program Effects %, Effect Size	*p* Value
1RM Bench (kg)	URT	38.7 (12.8)	45.2 (14.4)	+17%, ES = 0.48	0.001 **
	SRT	40.7 (10)	45.1 (10.4)	+11%, ES = 0.43	0.032 *
1RM Squat (kg)	URT	40.1 (16.8)	47.4 (15.1)	+18%, ES = 0.46	0.076
	SRT	43.4 (14.7)	54.9 (14.2)	+26%, ES =0.80	0.001 **
P Bench (W)	URT	174.8 (53.2)	213.2 (68.6)	+22%, ES = 0.63	0.001 **
	SRT	182 (65.2)	227.7 (79.9)	+25%, ES = 0.65	0.047 *
P Squat (W)	URT	252.5 (89.9)	275.4 (80.1)	+9%, ES = 0.27	0.253
	SRT	269.4 (119.1)	266.1 (86.7)	−1%, ES = 0.03	0.910
AB60 (reps)	URT	46.5 (7.4)	63.8 (7.1)	+37%, ES = 2.51	0.001 **
	SRT	53.7 (5.1)	63 (3.4)	+17%, ES = 2.15	0.001 **
SLJ (cm)	URT	188.1 (19.2)	196.6 (20.9)	+5%, ES = 0.40	0.010 *
	SRT	190 (27.5)	199.5 (26.5)	+5%, ES = 0.37	0.031 *

** = differences between groups; *p* < 0.001; * = differences between groups; *p* < 0.05. Abbreviations: 1RM = 1 repetition maximum; P = peak power; SLJ = standing long jump; W = watts; AB60 = abdominal muscle strength test; URT = unstable resistance training group; SRT = stable resistance training group; cm = centimeter; kg = kilogram; reps = repetitions; ES = effect size; Pre– = pretest; Post– = posttest.

## Data Availability

The data presented in this study are available upon request from the corresponding author.
